# Factors related to discontinued clinic attendance by patients with podoconiosis in southern Ethiopia: a qualitative study

**DOI:** 10.1186/1471-2458-12-902

**Published:** 2012-10-24

**Authors:** Abebayehu Tora, Gail Davey, Getnet Tadele

**Affiliations:** 1 Department of Sociology, Wolaita Sodo University, Sodo, Ethiopia; 2 Brighton and Sussex Medical school, University of Sussex, Sussex, UK; 3 Department of Sociology, Addis Ababa University, Addis Ababa, Ethiopia

**Keywords:** Podoconiosis, Factors, Treatment adherence, Ethiopia

## Abstract

**Background:**

Podoconiosis is a lymphoedema of non-infectious cause which results in long-term ill health in affected individuals. Simple, effective treatment is available in certain parts of Ethiopia, but evidence indicates that not all patients continue collecting treatment supplies from clinic sites once started. We used qualitative techniques to explore factors related to discontinued attendance at outreach clinics of a non-government organization in southern Ethiopia.

**Methods:**

A cross-sectional qualitative study was conducted in four clinic sites through unstructured in-depth interviews, key informant interviews and focus group discussions with the involvement of 88 study subjects.

**Results:**

Discontinuation of clinic visits is common among podoconiosis patients. The reasons were: remoteness from the clinic sites, unrealistic expectation of ‘special’ aid, worry about increasing stigma, illness and misconceptions about treatment.

**Conclusions:**

Several of these factors are remediable through community and individual information and education. Appropriate routes to deliver this information must be identified. Certain factors (such as distance to clinic sites and stigma) require substantial expansion of services or liaison with village-level government health services.

## Background

Podoconiosis is a form of geochemical non-filarial elephantiasis that results in bilateral lymphoedema of the lower legs [1]. It is common among poor farmers who have persistent barefoot contact with irritant red clay soil rich in silicate particles [2]. Areas of high prevalence of podoconiosis have been documented in tropical Africa, Central America and north India [3]. Of affected countries, Ethiopia appears to have the highest number of people with podoconiosis [4], with 11 million people at risk through exposure to irritant soil, and an estimated 1 million people affected countrywide [5]. As the disease progresses, patients become bedridden and unable to work [5,6]. Long term ill health results in a range of direct and indirect costs: medical and non medical expenses; the opportunity cost of time seeking healthcare; and the productivity cost of reduced working days [6].

In spite of these huge social and economic costs, podoconiosis remains relatively neglected in terms of policy response or public health intervention at national and international level [7]. Podoconiosis seems to be neglected because it is non-communicable and causes long term morbidity rather than mortality [5,8]. There is no mention of podoconiosis in the Ethiopian government’s 3^rd^ or 4^th^ Health Sector Development Plan (HSDP-III or HSDP IV) [8], and as yet no provision of prevention or treatment services through government health facilities.

A local non-governmental organization, the Mossy Foot Treatment and Prevention Association (MFTPA) was established specifically to provide treatment and care for patients with podoconiosis in Wolaita zone, an endemic area in southern Ethiopia [8]. Since its establishment in 1998, thousands of patients from the locality and surrounding zones have been treated at outreach clinic sites [8]. Although outreach clinics that provide health education and treatment to registered patients are open on a weekly basis, any individual patient is expected to attend only once per month for supervised treatment and to collect treatment supplies [8]. The clinic site health agents and social workers train patients to self-manage lymphoedema through careful washing with soap and water, use of an antiseptic, drying between toes and folds, use of moisturizing or antiseptic cream (e.g., Whitfield’s ointment), elastic bandaging in selected patients, elevation of the leg, controlled exercise and use of closed footwear and socks [8]. This regime has been found to be effective in improving disease stage, leg circumference and quality of life [9]. However, patients with advanced stage disease may not exhibit improvement overnight; lifetime care and treatment are important to control the progress of the disease and other complications [10].

Anecdotal evidence from clinic registers suggests that a proportion of registered patients with podoconiosis do not continue collecting treatment supplies from outreach clinics having started. Studies indicate that poor adherence to appropriate medication therapy for chronic conditions results in complications and increased health care costs [11,12]. In developing countries, like Ethiopia, when poor access to health care, possible lack of diagnosis, and limited availability of medications are taken into account, poor adherence threatens efforts to treat chronic conditions [13]. Complex socioeconomic, demographic and psychological factors have been reported in various studies as affecting adherence to medication therapy of chronic diseases [14-17]. According to Wood, the first step to overcome adherence challenges is to identify factors that prevent adherence [11]. Yet, no studies have been carried out to identify factors related to discontinued collection of treatment supplies by podoconiosis patients in Ethiopia. We therefore aimed to explore factors related to discontinued attendance at treatment and prevention clinics by patients with podoconiosis.

## Methods

### Ethics considerations

The study participants were recruited once they were fully aware of the purpose of the study and the methods of data collection. They were asked to provide witnessed oral consent to participation in the study. No written consent was asked for fear this might bias the interview process as most of the study subjects were illiterate [18]. Consent was witnessed by at least one person other than the principal investigator. For children under the age of 18, parents were asked to provide oral consent. Potential participants were clearly informed that they had a right to stop the interview at any time or to skip questions they did not want to answer. They were also told that interviews would be held in a private place and that identifiable data would not be viewed by any third party. Permission was also gained from the MFTPA since all of the study subjects were approached through the organization’s outreach clinics. The scientific committee in College of Social Sciences in Addis Ababa University approved the study based on the above ethical considerations.

### Study area

The study was conducted in Wolaita zone, southern Ethiopia, located 385kms from Addis Ababa, the capital of Ethiopia. Wolaita is one of the most densely populated areas in Ethiopia with an estimated total population of 1.7 million in 2007[19]. Most people earn their living from subsistence agriculture, and high prevalence of podoconiosis has been documented in this area [2]. The Mossy Foot Treatment and Prevention Association (MFTPA) offers community-based prevention and treatment at 15 outreach sites located 15 to 65km from the headquarters in Sodo town. Treatment supplies are offered every month free or for a small fee (2 Birr, £0.08 or $0.12).

### Study design and sampling

We conducted a qualitative study using in-depth-interviews (IDIs), focus group discussions (FGDs) and key informant interviews (KIIs). Four of fifteen clinic sites of the Mossy Foot Treatment and Prevention Association (MFTPA) were purposively selected to include a wide range of patient follow-up durations.

We employed theoretical sampling techniques to recruit study subjects into In-depth Interviews (IDI) and Focus Group Discussions (FGD). Forty-four podoconiosis patients took part in In-Depth Interviews. People who came to the clinic sites for purposes other than collecting treatment supplies were excluded. The MFTPA project director and social work department head participated in Key Informant interviews as they have established extensive relationships with the patients. Having identified FGD participants with the help of clinic site staff, we grouped them into three categories: community and religious leaders, podoconiosis patients, and clinic site health agents and social workers. Two FGDs with male community and religious leaders, three FGDs with podoconiosis patients (one for women only, two mixed), and two FGDs with female and male clinic site health agents and social workers were held. Each group was composed of six participants giving a total of forty two participants. Data collection continued until no new information emerged through further interviews.

### Data collection

Data collection was conducted over a four week period in March 2010 by AT (a native speaker of the local language, *Wolaitigna*). Three types of unstructured and pilot tested interview guides were used to conduct Focus Group Discussions (FGDs), Key Informant Interviews (KIIs), and In-depth interviews (IDIs). In-depth interview informants were approached on the days scheduled for clinic visits and asked to indicate whether they had missed a visit at least once since their first visit. Each month, each patient needs to attend clinic for education and treatment supplies. We therefore defined any patient not attending clinic on one or more scheduled occasion(s) as non-adherent. Those who had missed collecting treatment supplies were interviewed to establish what factors might have contributed. Clinic site staff arranged a private setting where interviews were held with each informant for about 20 to 30 minutes. Information obtained through in-depth interviews was complemented by key informant interviews and focus group discussions. Focus group discussion participants were asked why they thought patients might not consistently attend clinic, while key informants and clinic site health agents were also asked how consistent patients were in collecting treatment. Discussions in all focus groups were held in neighboring homes where only the recruited participants gathered for about 1 and half hours. An experienced note-taker was hired to take notes during individual interviews and focus group discussions. Audio recording was also used with prior consent of the informants. A unique identifying number was given to all informants in advance to link the data obtained through interviews and focus group discussions.

### Data analysis

AT transcribed field notes and audio files of interviews and focus group discussions in the local languages (both Amharic and Wolaitigna) used during data collection and checked for accuracy through replay of cassettes and revision of field notes. Unique identifying numbers and demographic variables assigned to each informant during data collection were linked to the data during transcription and names of specific people mentioned in the data were removed. A meaning based translation of transcripts was made by AT and GT. The text data typed into the word file were organized using manual coding techniques to code and categorize similar ideas. To increase the validity of coding, three coders were involved to organize the data based on themes of interview guides and themes derived from the data. The coders resolved overlapping themes and inconsistencies in data sources through regular meetings, and incorporated additional themes suggested in the discussion.

In the coding process, informants who had missed collecting treatment supplies were categorized as ‘irregular visitors’ for the purpose of further analysis. It has been suggested that identification of non-adherent patients is of particular importance because they represent the highest priority target of intervention strategies [20]. Themes identified in interviews and focus group discussions through questions about reasons for irregular visits were categorized as determinants of discontinued attendance.

## Results

Eighty-eight participants were included; forty male and forty-eight female. Participants were aged between 16 and 75 years. The following factors appeared to be related to discontinuation of clinic visits among podoconiosis patients.

### Remoteness from the clinic site

Distance of clinic sites from patients’ homes, coupled with physical disability and difficulty finding and affording transport, are prime reasons for non-adherence.

"I began treatment when it was being provided in a town named Badesa. It took me three hours on foot to get there. I tried to visit the clinic for some time but stopped when my feet developed wounds***[In-depth interview informant, a 30 year old female patient***]."

According to some FGD participants, there are no modern forms of transport in the villages in which podoconiosis patients live. Patients living in such areas are forced to walk long distances on foot, making attendance very difficult.

"There are villages where there are no means of transport. The only alternative for patients in such areas is to walk on foot. But, to walk long distances for a podoconiosis patient is challenging. Thus, some patients refrain from visiting the clinic sites located far from their residence***[FGD participant, religious leader, 30 years].***"

Even if transport is available, patients may be unable to afford it.

"I sometimes postpone visiting the clinic when I have no money for transport. I avoid walking on foot fearing wounds that may develop. When I walk, my foot immediately develops wounds, so I remain at home in such conditions***[In-depth interview informant, 16 years].***"

Some of the FGD participants thought the problem of distance was even greater for older patients.

"A lot of older patients stop visiting the clinics. They find it difficult to walk long distances. Some older patients send their children or other individuals to the clinics***[FGD participant, 50 years].***"

Responses from the study subjects indicate that the existing services are not accessible enough to patients living in remote places. Several stated that expansion of services to remote rural villages was likely to improve clinic attendance.

### Expectations of ‘special’ support

MFTPA staff thought that some patients came to the clinic sites expecting aid in the form of material or financial benefits other than treatment supplies from the organization. Such patients visit the clinic once or twice and stop after realizing that there is nothing ‘special’ beyond the treatment program.

"Some patients think that they deserve special support just because they are patients. Such patients stop visiting the clinic after realizing that there is no special benefit. They don’t even consider that they are getting the treatment for their own health***[FGD participant, clinic site health agent, 34 years].***"

The misconception that other patients who make regular visits get special benefits from the non-government organization is a ‘pull’ factor for some. Patients who begin with these misconceptions soon stop once they realize that there is no special advantage.

"Once, I approached a podoconiosis patient who had stopped receiving clinic treatment. I asked him to begin treatment again. He replied ‘You wouldn’t knock at everybody’s door unless you received some special privilege in our name’. I was shocked when the man reacted in such a way***[FGD participant, clinic site social worker, 28 years]***."

Seeking special aid becomes particularly common among patients during times of drought or famine. At these times, patients fail to make regular clinic visits. According to the MFTPA Social Work Department head, patients openly ask the organization to provide them food and other forms of aid in such hard times.

"Patients expect something from the organization during drought and famine. During these seasons it is quite difficult to provide only treatment supplies while patients are literally suffering from starvation. As providing food items and other aids is not the role of this organization, some patients abandon visiting the clinics until they pass those hard times***[Key informant, head of MFTPA’s social work department].***"

### Worry about increasing stigma

Podoconiosis patients are deterred from seeking treatment because they fear this will reinforce stigma against them. Since the clinics at which treatment is offered are for podoconiosis patients only, some patients fear that attendance will identify them as having the disease.

"I was making house visits that day. I heard that there was a child who had stopped attending the clinic because his parents did not wish him to attend. I asked his parents why they prevented the child from visiting the clinic. Their immediate response was ‘there is nobody sick in our home’. What I understood from their response is that they feared being identified as having a podoconiosis patient in their family***[FGD participant, community leader***,***32 years]***."

Worry about increasing stigma also affects the health seeking behavior of patients with early manifestations. Patients with early manifestations avoid visiting the clinic sites in order to avoid public identification. These patients do not want to be identified as having podoconiosis. In the early stages, they hide their feet by wearing long clothes and shoes, and only start seeking treatment once the swelling becomes difficult to disguise.

"When we tell some patients with early manifestations of podoconiosis to come to clinic, they respond abruptly that they are not sick. They don’t like to be pointed out as “gedya kita” [local name for a swollen foot]. I know a lot of patients who neglected our warnings but later started to receive treatment after the swelling had progressed to more advanced stages***[FGD participant, clinic site health agent, 30 years]***."

Some family members prevented children or other dependents from using the treatment supplies they collected from the MFTPA outreach clinics because of the discomfort they feel about the smell.

"I live with my aunt. Once, she said ‘all your feet, your shoes, and the medicine you use are stinky’. It suffocates the house. She thought it is a big humiliation when people enter the house. She then threw both my shoes and the bleach outside. As a result, I was discouraged to receive treatment kits [***In-depth interview informant, 18 years].***"

### Illness

Recurrent illness prevents patients from attending clinic regularly. Patients become ill with acute adenolymphangitis (ALA): attacks of fever, pain and increased swelling of the leg related to super-infection, physical injuries or strenuous activity. During such an attack they may become bedridden for days or even weeks. Consequently, they cannot attend clinic and thus do not collect supplies necessary for treatment.

"I remember it was when I was coming from the market. I went to the market to sell something. It was very hot and sunny. I was standing long hours in that situation. Finally, when I was coming home I fell down in the middle of the road. People gathered together, carried me and took me to my home. I was completely unconscious when all that happened. I stayed on bed for weeks until I recovered***[FGD participant, clinic site health agent, 32 years].***"

"Walking long distances and working long hours on the farm is not good. It brings big swelling in ‘ankakuleta’ [a local name for swelling of lymph nodes] and blocks any movement, and then brings headache and shivering. The illness may stay for weeks and prevent collection of treatment supplies [***In-depth interview informant, 35 years].***"

Injuries of the foot also cause illness to the patient. A 28 year old in-depth interview informant indicated why he could not consistently collect treatment supplies.

"I missed clinic for four consecutive months. I was cut by a sharp thing while I was working on the farm. It took four months for me to recover. At that time I had no-body to send to the clinic to bring the treatment kits. The pain and the swelling worsened***.***"

### Misconceptions about treatment

According to FGD participants, patients with podoconiosis make great efforts (using both traditional and modern forms of treatment) to treat their feet. If, despite their effort and expense, they do not see improvement using one form of treatment, some doubt that any treatment will be effective. According to a FGD participant,

"There is a person who I know in my village. He has been suffering from the disease for many years. He went to many places looking for holy water and went to several hospitals, but did not see any improvement. His foot was getting worse whatever he did. Once, I informed him about the podoconiosis treatment. But, he would not believe that his foot might recover. After repeated visits to his home to tell him about the treatment, I managed to bring him to the clinic site so that he saw some improvement after consistent follow-up***[FGD participant, 42 years].***"

Failure to experience immediate improvement after receiving podoconiosis treatment may also make some patients stop attending. Some in-depth interview informants indicated that they had stopped visiting the clinics thinking that the treatment was not effective.

"I stopped going to the clinic site when I couldn’t see any improvement. I was eager to get normal feet back. But it did not happen. But the condition of my foot deteriorated after I stopped receiving treatment supplies***[In-depth interview informant, 22 years].***"

Even though patients are consistently advised that they need to stick with the lymphoedema treatment regimen, some patients still discontinue treatment. According to a clinic site agent, “*most patients come to the clinic sites expecting to get immediate relief, so they become disappointed and stop receiving treatment when they fail to experience improvement*”.

In contrast, there are also some podoconiosis patients who experience improvement soon after starting treatment. Such patients think their feet are healthy again, and stop attending. They may only restart treatment when their feet deteriorate and develop increased swelling, wounds, or mossy changes.

"I stopped taking the treatment kits thinking that my foot was cured. After four months without treatment, my foot started to swell up again. I started the treatment again after I experienced further complications***[In-depth interview informant, 30 years]***."

Such patients are misled by the improvement they experience after following treatment for a short time. The clinic site agents stated that they repeatedly warn the patients not to stop treatment irrespective of improvement as the disease may progress due to further exposure to the environmental trigger. According to the clinic site health agent,

"Some patients come to the clinic site when they get sick but stop after seeing some improvement. We teach them not to quit the treatment by stressing that the disease needs lifelong care and treatment.***[FGD participant, 34 years].***"

## Discussion

In Wolaita zone where over 5% of the total population is estimated to have lymphoedema secondary to podoconiosis [5], the MFTPA is the only source of education on lymphoedema management and treatment supplies. The MFTPA uses a model of community-based care led by expert patients and supported by community Network Groups [8]. Even though recent work in northern Nigeria [21] identified a similar model (community-selected and supported care-givers) as the most effective in reducing lymphoedema and adenolymphangitis episodes, it has been clear in Wolaita that not all podoconiosis patients continue treatment. We discuss here some of the factors that appear to reduce patients’ adherence with attending a clinic to collect podoconiosis treatment supplies. Five key factors were identified: distance to the clinic sites, unrealistic expectation of ‘special’ aid, worry about increasing stigma, illness, and misconceptions about treatment. However, as a qualitative study, our aim was exploratory, and we could not measure the variation in influence of these factors by sociodemographic characteristics or level of disease severity.

First, the locations of the MFTPA clinics may be inaccessible for patients who live in remote rural villages. Most clinics are located in small towns, while the large majority of podoconiosis patients are from the surrounding rural areas [2,5,8]. These areas often lack means of transport, so patients are forced to walk long distances on foot, which is challenging because of the disability caused by their foot swelling. Even if there is transport, it may be unaffordable to patients. Similar barriers have been described in relation to treatment for lymphatic filariasis (LF) in northern Nigeria [21] and in Kenya [22]. While it may be impossible for a small NGO like MFTPA to increase accessibility through expansion, linking podoconiosis treatment with government services delivered at village level may improve accessibility for patients.

Second, as most patients with podoconiosis are very poor rural farmers [1,7] whose disease has exacerbated their poverty [6], they will naturally look for other forms of support in addition to treatment. In this study, we found that some podoconiosis patients discontinue collecting treatment supplies, particularly during drought or famine, because they are not offered financial or material support other than treatment supplies. This may be because most Non-Governmental Organization (NGOs) working in rural areas have broader development aims than the MFTPA which focuses on control of one specific disease. Many podoconiosis patients may have experience of receiving more direct financial or other forms of support, creating unrealistic expectations of what the MFTPA might offer. The MFTPA must improve community awareness of its specific role in podoconiosis control, and increase collaboration with other agencies so that patients requiring other forms of assistance can be referred appropriately. Other governmental and non-governmental organizations should be invited to engage in (re)building the livelihoods of such patients.

Third, perceived stigma from family members or the wider community is a serious barrier to adherence with collecting clinic based lymphoedema treatment supplies, and is manifested in avoidance of the treatment clinics as a coping strategy [23]. Provision of treatment supplies through MFTPA clinics, which the public know are for podoconiosis patients only, generates deep fear of identification among patients. Patients in the early stages of disease who disguise it find this particularly difficult, and may either completely avoid the clinics, or may start collecting treatment supplies and then stop. A study on lymphatic filariasis showed that perceived stigma was an important issue in causing delay in diagnosis and treatment of patients [24]. Studies on determinants of adherence of people living with HIV to Antiretroviral Treatment (ART) also confirm that lack of social support and discrimination by family members contribute for dropout of treatment despite improved health and interest of the patient [14-16]. Integration of podoconiosis treatment with other primary health care services may be effective in tackling stigma related to accessing treatment from existing vertical or stand-alone program.

Fourth, although lymphoedema care reduces acute adenolymphangitis episodes in LF [21] and anecdotally also in podoconiosis, if these episodes occur despite treatment, they may themselves prevent patients attending clinic for treatment and further supplies. Adenolymphangitis may be triggered by circumstances such as strenuous activity, walking long distances, and cold or rainy seasons. More flexible systems for distribution of treatment supplies may be necessary to take into account patients who develop acute illness and are unable to attend clinic.

Fifth, some podoconiosis patients stop collecting supplies because of misconceptions about the treatment regimen. While some discontinue because of the assumption that the treatment is not working, others stop because they feel healthy after receiving treatment for some time. Once patients reach the advanced stages of podoconiosis, many of the changes that have occurred are irreversible [18]. Treatment prevents further progression and diminishes episodes of acute adenolymphangitis [9], but will not achieve reversal of all the swelling and dermal overgrowth that are prominent in late stage disease [10]. Discontinuation because of disappointment in results was also reported in LF treatment study [21]. The more advanced the stage of disease, the more likely patients are to doubt the effectiveness of treatment since improvement is rarely experienced. Conversely, some podoconiosis patients discontinue collecting treatment supplies because they experience substantial improvement in the condition of their feet.

## Conclusion

In conclusion, this study has identified a range of factors that determine adherence of patients with collecting treatment supplies from MFTPA clinics. Some of these factors can be overcome with relatively simple measures: improving patient understanding of the need for lifelong treatment; better informing patients about the likely outcomes of podoconiosis treatment; and convincing patients of the need to prioritize treatment over other activities. Reducing distance to a clinic or supply distribution point will require linking MFTPA services with government health schemes in villages, and reducing community stigma against patients will require a multi-agency approach and considerable time.

## Competing interests

The authors declare that they have no competing interests.

## Authors’ contributions

AT conceived the study design, conducted data collection, analyzed and interpreted the data and prepared draft of the manuscript. GD and GT advised during the study design, participated in analysis and interpretation of the data and reviewed the manuscript. All authors read and approved the final manuscript. GD is guarantor of the paper.

## Pre-publication history

The pre-publication history for this paper can be accessed here:

http://www.biomedcentral.com/1471-2458/12/902/prepub
